# Maximum Sprints Elicit Higher Peak Knee Joint Power than Resistance Training Exercises

**DOI:** 10.3390/sports14060221

**Published:** 2026-05-28

**Authors:** Tobias Alt, Kenneth P. Clark, Jesper Augustsson, Jurdan Mendiguchia

**Affiliations:** 1 Department of Biomechanics, Performance Analysis and Strength & Conditioning, Olympic Training and Testing Centre Westphalia, 44139 Dortmund, Germany; tobias.alt@osp-westfalen.de; 2 Department of Kinesiology, West Chester University of Pennsylvania, West Chester, PA 19383, USA; kclark@wcupa.edu; 3 Department of Sport Science, Faculty of Social Sciences, Linnaeus University, 352 52 Växjö, Sweden; 4 Department of Physical Therapy, ZENTRUM Rehab and Performance Center, 31010 Barañain, Spain; jurdan24@hotmail.com

**Keywords:** strength, sprint mechanics, eccentric training, hamstrings

## Abstract

Background: Sprinting places exceptionally large mechanical demands on the knee flexors, particularly during the late swing phase when the hamstrings are actively lengthened under load. Therefore, coaches and practitioners try to increase the hamstrings’ capacity to absorb energy by various resistance training exercises with high (supramaximal) eccentric intensity. However, it is unclear whether the load parameters are equivalent to maximum sprints. Consequently, the aim of this short study was to compare peak knee power values derived from aggregated previously published datasets of maximum sprints and strenuous hamstring exercises, rather than from a single directly controlled experimental comparison. Methods: Previously published inverse dynamic analyses of sprints, explosive Razor Curls, decelerated Nordic Hamstring Exercise and eccentric isokinetic hamstring tests were aggregated and compared. Results: The combined data showed that both absolute and relative peak knee power were 8 to 14 times higher during sprinting at 10 m/s—primarily due to 6- to 14-fold higher knee extension angular velocities. Conclusions: Peak knee power during maximum sprints was not replicated by the analyzed hamstring exercise conditions, even if they were very explosive and strenuous.

## 1. Introduction

The hamstrings, in addition to playing a major role in sprinting, are the most prevalent non-contact injury location in high-speed running sports such as track and field, soccer, basketball, baseball, American and Australian football and rugby [1,2,3]. According to most prior research, the late swing phase of the sprint cycle—where the hamstring muscles are significantly activated—is considered the phase during which injury most likely occurs [4]. During this part of the cycle, the posterior thigh muscles, in particular the hamstrings, generate tension while lengthening to decelerate knee extension and absorb and redistribute the kinetic energy of the swing limb before foot contact [5]. Concretely, the hamstring muscle fibers undergo lengthening from approximately 45% to 90% of the sprinting gait cycle (i.e., swing phase) absorbing the imposed mechanical energy, and thereafter the muscle-tendon units, and hence the tendon stretches and stores energy to reuse at initial contact (i.e., foot strike) [5,6]. In summary, the hamstring muscles likely function as a shock absorber in series with a spring that cyclically absorbs and recovers elastic energy before foot contact during the sprinting gait cycle [7].

This ability of the knee flexors to absorb energy during the late swing phase of the sprint cycle—inter alia by the hamstrings—has received surprisingly little attention from the scientific community [8] although it was suggested as a key determinant of both hamstring strain injury risk [5,6] and sprint performance [9]. The ability of the hamstrings to function as a shock absorber can be physically represented by the negative work exerted by the knee flexors in order to counteract the opposite direction of the tibia movement (knee extension) as occurring during the late swing phase. For explosive movements, the knee joint power is better suited because it takes time (P = W/t) or rather the actual movement velocity (P = M × ω) into account. Although peak knee joint power is a joint-level surrogate and not a direct measure of hamstring muscle or tendon loading, peak knee joint power alone explained 36% of the variance in maximum sprint speed in trained male adolescent sprinters [8]. Pioneering basic laboratory studies conducted by Garrett et al. [10,11] pointed to the ability to absorb energy, compared to total strain and force, as a key factor to consider when an active muscle is stretched until failure. In short, the greater the absorption, the greater the protection for an actively elongated muscle, similarly during maximal sprints, where the posterior thigh muscles are activated [6,12]. Musculoskeletal models showed that the energy absorption capacity expressed as negative work of the hamstring during the late swing phase increased in greater proportion than strength when athletes increased sprinting speed from 80% to 100% without any change on peak musculotendinous length [6]. Based on the seminal work of Chapman and Caldwell (1983) [9] on the swing-based “energy management” theory, it has been demonstrated that the maximum sprint speed is limited by the ability of the knee flexors to reduce the kinetic energy of the lower limb while they lengthen in late swing, implicating the eccentric force–velocity relationship [13]. Recently published hamstring prevention frameworks [7,14] suggest that greater hamstring energy absorption capacity may increase both resistance to injury and tolerance to higher sprinting speeds, potentially also during repeated sprints. Thus, eccentric hamstring exercises are recommended. Holistic prevention programs usually include the Nordic Hamstring Exercise (NHE), the Razor Curl (RC), and isokinetic exercise (Iso) [15]. These conditions were selected because they represent commonly used high-intensity eccentric hamstring exercises for which previously published datasets permitting calculation of peak knee joint power were available.

**Figure 1:**
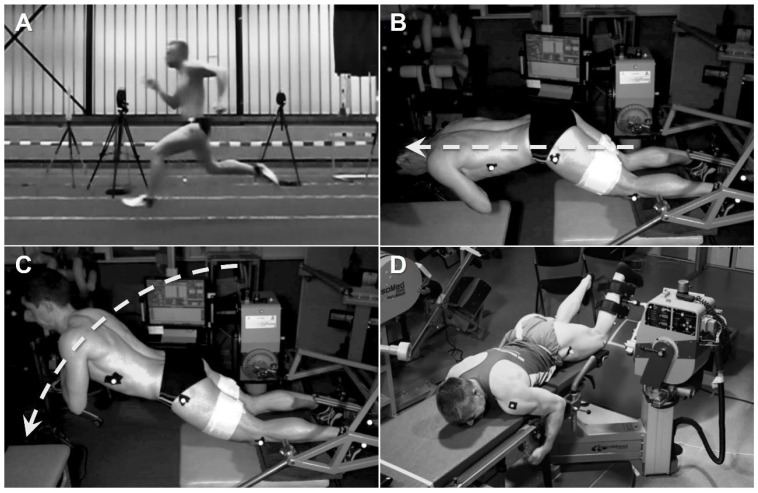
Overview of the four analyzed hamstring loading conditions: (**A**) maximum velocity sprinting, (**B**) explosive eccentric-only Razor Curl, (**C**) decelerated Nordic Hamstring Exercise, and (**D**) eccentric isokinetic hamstring testing at 150°/s. All tasks impose high to maximal loads on the hamstring muscle–tendon unit. The photographs illustrate knee and hip joint configurations at the instant of peak knee joint power. Panels (**A**,**D**) reproduced from Alt et al. [8] with permission; panels (**B**,**C**) are original to the present submission.

To the best of the authors’ knowledge, no previous study has aggregated and compared peak knee joint power values from the selected previously published datasets included in the present communication. Consequently, the purpose of the current short communication was to compare peak knee joint power values derived from selected previously published datasets [8,16,17] involving maximal velocity sprinting and strenuous high-velocity eccentric hamstring exercise conditions (Figure 1).

## 2. Materials and Methods

The data were aggregated from previously published work where the exact methods can be found [8,16,17]. The present communication did not involve a new reanalysis of original raw data across all conditions. Instead, previously published peak knee joint power values were compiled and descriptively compared in conjunction with kinematic and kinetic data from selected datasets.

### 2.1. Sprinting and Isokinetics

Twenty-two male sprinters (mean age: 22 years; height: 181 cm; body mass: 77 kg) performed maximal sprints (~9.8 m/s) on an indoor track. Kinematic data were collected using a 10-camera (100 fps) 3D motion capture system (VICON MX40, Vicon Motion Systems Ltd., Oxford, UK) with 28 retroreflective markers. Sprint analyses were processed using VICON Nexus (version 2.3; Vicon Motion Systems Ltd., Oxford, UK) [8]. Sprint analyses were based on the biomechanical model described by Lund et al. [18]. Segment coordinate systems were defined according to the ISB recommendations for reporting joint kinematics [19]. The knee joint was modeled according to the joint coordinate system approach proposed by Grood and Suntay [20]. Joint rotations were calculated using a Z–X–Y Cardan sequence under standard ISB coordinate axis definitions, corresponding to flexion/extension, abduction/adduction, and internal/external rotation, respectively.

Eccentric knee flexor strength was assessed with an IsoMed 2000 isokinetic dynamometer (D&R Ferstl GmbH, Hemau, Germany) at 150°/s through a 110° range of motion in the prone position. Data were recorded at 200 Hz and synchronized with high-speed video (100 fps) for motion tracking (camera-based knee angular velocity) using VICON Peak Motus (version 10.0.1, Vicon Motion Systems Ltd., Oxford, UK) [16].

### 2.2. Nordic Hamstring Exercise and Razor Curl

One experienced male athlete (aged 33, height 171 cm, body mass 69 kg) performed NHE_dec_ and RC_exp_ trials. For each condition, three repetitions were analyzed. Thus, unlike the sprinting and isokinetic datasets, which were based on the group-level participant samples (*n* = 22), the RC_exp_ and NHE_dec_ data represent repeated trials from a single participant. A custom frame enabled unilateral isokinetic torque measurements using an IsoMed 2000 dynamometer (D&R Ferstl GmbH, Hemau, Germany), synchronized with high-speed video (100 fps) and motion capture. Markers were placed on key anatomical landmarks and on the dynamometer lever. Kinematic data were analyzed in VICON Peak Motus (version 10.0.1, Vicon Motion Systems Ltd., Oxford, UK) [17].

## 3. Results

Table 1 lists relevant kinematic and kinetic values of maximal velocity sprints (9.8 ± 0.5 m/s), explosive eccentric-only Razor Curls (RC_exp_), decelerated Nordic Hamstring Exercise (NHE_dec_) and eccentric isokinetic hamstring tests at 150°/s in a prone position (Iso_Hecc150_). Within the analyzed datasets, absolute and relative peak power was 8.1 to 13.6 times higher during sprinting compared with the eccentric hamstring exercise conditions. Eccentric knee joint power is driven by explosive hip extension [8], a mechanism which was not replicated by the resistance training exercises analyzed here [15] (Table 1). Even the explosive Razor Curl as a multi-joint exercise with strenuous difficulty (most elite athletes are not able to perform it in the investigated fashion) remained far below sprinting hip kinematics and even further below sprinting knee extension velocity (6.3 times higher in sprinting).

## 4. Discussion

Despite the widespread use of eccentric hamstring exercises and maximal-velocity sprints in performance training, no studies have directly compared their peak power outputs. Consequently, it remains unclear whether the loading characteristics of commonly used hamstring exercises approximate those experienced during maximal sprinting. Therefore, the present short study sought to synthesize and compare peak power values reported in previously published studies.

If a part of the prevention strategy is aimed at increasing the energy absorption capacity of the hamstrings in order to protect against injury and improve sprinting performance, training should likely include conditions involving very high tibial angular velocities. Such conditions may enhance the hamstrings’ ability to absorb mechanical energy during high-speed running. However, the authors want to emphasize that the positive effects generated by commonly used eccentric exercises, which are typically performed at substantially lower movement velocities than sprinting, should not be neglected by the presented results (optimum length, collagen synthesis, fascicle length, tendon stiffness, etc.) [21,22].

Strength is typically linked to increasing external load (F = m × a), which demands large muscular forces but reduces movement velocity and acceleration. In contrast, enhancing hamstring power and negative work relies more on maximizing acceleration in this equation, and—analogous to the attempts to relate the hamstring electromyographic activity during sprinting to strength exercises—no single exercise was capable of replicating the movement-specific knee angular velocities (~1000°/s) [7,8] (Table 1) or the levels of activation observed during sprinting [23].

Interestingly, the peak moments of the four conditions (sprint vs. three hamstring exercises) demonstrated the narrowest range (<1.5× difference). This fact emphasizes that, among these exercise conditions, the amount of peak power primarily depends on the knee angular velocity. Besides the large differences in knee kinematics (6.3 to 14.0 times higher extension velocity in sprints), the hip extension angular velocity and hip flexion angle at the instance of peak knee joint power significantly diverged (Table 1).

Therefore, the present findings may suggest that maximum velocity sprinting and variants such as wearable resistance (e.g., sleeves providing small additional tibial loads) could increase the hamstrings’ mechanical energy absorption demands. This quality may be important both for tolerating high-speed sprinting and for reducing susceptibility to hamstring tissue damage.

The present comparison suggests combining slow and fast eccentric movements in the strength component of a holistic preventive program [15], since they may target different qualities and properties of the hamstring muscles. It is important to mention that common hamstring exercises (i.e., eccentric-only, relatively slow movement speed) are neither designed to mirror the nature of sprinting (explosive plyometric action) nor specific injury mechanisms, but rather to prepare the involved tissues for the specific loading characteristics. Future research is required to understand the role of hip flexion and pelvis position on hamstring mechanics [14,17] as well as how their tendons adapt most effectively. This is important because the ability to generate and withstand large forces at high movement speeds is founded on a complex interaction between muscles and tendons [7]. Peak knee joint power is driven by an explosive sequence of hip and knee extension (“whip from the hip”) [7,8,24] exceeding those observed in the resistance training exercises analyzed here. Nonetheless, it is important to notice that a solid foundation and intense long-term preparation (especially for the tendons) is strongly needed prior to performing fast eccentric movements.

Ultimately, sprinting involves far more than peak knee joint power. It represents a complex neuromuscular interaction between the stance leg and swing elements, characterized by rapid sequences of activation and relaxation [14,23].

The authors are aware of the limitations of the aggregated datasets from heterogeneous sources (e.g., unequal sample sizes, single-athlete data), but they want to emphasize the insights which may be provided by this procedure. For greater internal validity and more robust conclusions, future studies should aim for data acquisition in a larger sample across all test conditions. Furthermore, the present communication did not include within-subject cross-condition testing and therefore cannot establish strict exercise equivalence or non-equivalence between conditions.

## 5. Conclusions

Coaches and practitioners should be aware that peak knee power observed during maximal-velocity sprints is unlikely to be fully replicated by the currently used resistance training exercises, even those that are highly explosive and demanding. Therefore, high-intensity sprint practice may contribute to the development of healthy, robust, and resilient hamstrings. However, the complementary benefits of eccentric resistance exercises (e.g., fascicle length, tendon stiffness, collagen synthesis) are highly important for holistic hamstring health and performance. Controlled eccentric exercises are especially essential for younger athletes and during rehabilitation to gradually increase the loading capacity.

## Figures and Tables

**Table 1:** Relevant sagittal plane knee and hip kinematic and kinetic values of maximal velocity sprints (9.8 ± 0.5 m/s), explosive Razor Curls (RC_exp_), decelerated Nordic Hamstring Exercise (NHE_dec_) and eccentric isokinetic hamstring tests at 150°/s (Iso_Hecc150_). The highest values are highlighted in gray.

	Wmax_knee_ [P]	Wmax_knee_rel [P/kg]	Mmax_knee_ [Nm]	Mmax_knee_rel [Nm/kg]	Mmax@Wmax [Nm]	Mmaxrel@Wmax [Nm/kg]	ωKE@Wmax [°/s]	φKF@Wmax [°]	ωHE@Wmax [°/s]	φHF@Wmax [°]
Sprint (*n* = 22)	3914 ± 806	51 ± 10	209 ± 26	2.6 ± 0.3	186 ± 32	2.3 ± 0.4	1206 ± 122	50 ± 5	688 ± 106	80 ± 6
RC_exp_ (*n* = 1; 3 trials)	438 ± 63	6 ± 1	204 ± 7	3.0 ± 0.1	131 ± 16	1.9 ± 0.2	192 ± 8	34 ± 4	332 ± 16	56 ± 2
NHE_dec_ (*n* = 1; 3 trials)	288 ± 48	4 ± 1	233 ± 13	3.4 ± 0.2	191 ± 9	2.8 ± 0.1	86 ± 10	17 ± 5	−10 ± 2	21 ± 5
Iso_Hecc150_ (*n* = 22)	300 ± 57	4 ± 1	168 ± 25	2.1 ± 0.3	154 ± 30	2.0 ± 0.3	112 ± 10	41 ± 10	−1 ± 5	12 ± 5

Abbreviations. W = joint power; M = joint moment; rel = relative to body mass; ω = joint angular velocity; φ = joint angle; KE = knee extension; KF = knee flexion; HE = hip extension; HF = hip flexion; RC = razor curl; exp = explosive; NHE = Nordic Hamstring Exercise; dec = decelerated; Iso = isokinetic; Hecc = eccentric hamstring test; 150 = isokinetic angular velocity.

## Data Availability

The raw data supporting the conclusions of this article will be made available by the authors on request.
